# Constitutive phosphorylated STAT3-associated gene signature is predictive for trastuzumab resistance in primary HER2-positive breast cancer

**DOI:** 10.1186/s12916-015-0416-2

**Published:** 2015-08-03

**Authors:** Amir Sonnenblick, Sylvain Brohée, Debora Fumagalli, Delphine Vincent, David Venet, Michail Ignatiadis, Roberto Salgado, Gert Van den Eynden, Françoise Rothé, Christine Desmedt, Patrick Neven, Sibylle Loibl, Carsten Denkert, Heikki Joensuu, Sherene Loi, Nicolas Sirtaine, Pirkko-Liisa Kellokumpu-Lehtinen, Martine Piccart, Christos Sotiriou

**Affiliations:** Breast Cancer Translational Research Laboratory J-C Heuson, Institut Jules Bordet, Bld de Waterloo, Université Libre de Bruxelles, 1000 Brussels, Belgium; Medical Oncology Unit, Institut Jules Bordet, Université Libre de Bruxelles, Bld de Waterloo, 1000, Brussels, Belgium; Molecular Immunology Lab, Institut Jules Bordet, Université Libre de Bruxelles, 1000 Brussels, Belgium; Multidisciplinary Breast Center, KULeuven, University Hospitals, Leuven, Belgium; German Breast Group, Neu-Isenburg and Sana-Klinikum, Offenbach, Germany; Institute of Pathology, Charité Hospital Campus Mitte, and German Cancer Consortium (DKTK), Berlin, Germany; Department of Oncology, Helsinki University, Hospital and Helsinki University, Helsinki, Finland; Division of Cancer Medicine and Research, Peter MacCallum Cancer Centre, East Melbourne, VIC Australia; Pathology Department, Institut Jules Bordet, Université Libre de Bruxelles, Brussels, Belgium; Department of Oncology, University of Tampere and Tampere University Hospital, PO Box 607, FIN 33101 Tampere, Finland

**Keywords:** Breast cancer, FinHer, HER2, Phosphorylated STAT3, Randomised trial, Trastuzumab resistance

## Abstract

**Background:**

The likelihood of recurrence in patients with breast cancer who have HER2-positive tumors is relatively high, although trastuzumab is a remarkably effective drug in this setting. Signal transducer and activator of transcription 3 protein (STAT3), a transcription factor that is persistently tyrosine-705 phosphorylated (pSTAT3) in response to numerous oncogenic signaling pathways, activates downstream proliferative and anti-apoptotic pathways. We hypothesized that pSTAT3 expression in HER2-positive breast cancer will confer trastuzumab resistance.

**Methods:**

We integrated reverse phase protein array (RPPA) and gene expression data from patients with HER2-positive breast cancer treated with trastuzumab in the adjuvant setting.

**Results:**

We show that a pSTAT3-associated gene signature (pSTAT3-GS) is able to predict pSTAT3 status in an independent dataset (TCGA; AUC = 0.77, *P* = 0.02). This suggests that STAT3 induces a characteristic set of gene expression changes in HER2-positive cancers. Tumors characterized as high pSTAT3-GS were associated with trastuzumab resistance (log rank *P* = 0.049). These results were confirmed using data from the prospective, randomized controlled FinHer study, where the effect was especially prominent in HER2-positive estrogen receptor (ER)-negative tumors (interaction test *P* = 0.02). Of interest, constitutively activated pSTAT3 tumors were associated with loss of PTEN, elevated IL6, and stromal reactivation.

**Conclusions:**

This study provides compelling evidence for a link between pSTAT3 and trastuzumab resistance in HER2-positive primary breast cancers. Our results suggest that it may be valuable to add agents targeting the STAT3 pathway to trastuzumab for treatment of HER2-positive breast cancer.

**Electronic supplementary material:**

The online version of this article (doi:10.1186/s12916-015-0416-2) contains supplementary material, which is available to authorized users.

## Background

Trastuzumab is a remarkably effective therapy for patients with HER2-positive breast cancers, both in the metastatic and adjuvant settings [1–3]. However, not all women with tumors expressing high levels of HER2 respond to trastuzumab. Several potential mechanisms to explain trastuzumab resistance have been proposed, including loss of PTEN, excessive signaling through the insulin-like growth factor-I receptor, or expression of p95HER2, a truncated form of the HER2 receptor that has kinase activity but lacks the extracellular trastuzumab-binding domain [4, 5].

Signal transducer and activator of transcription proteins (STATs) are latent cytoplasmic proteins that form functional dimers with each other when activated by tyrosine phosphorylation, upon which they translocate to the nucleus to regulate the expression of genes by binding to specific elements within gene promoters. STAT3 appears to be a point of convergence for numerous oncogenic signaling pathways [6]. Indeed, constitutive activation of STAT3 has frequently been detected in diverse human cancer cell lines and tissues, including breast cancers [6]. Many studies have suggested that STAT proteins could participate in oncogenesis by up-regulating genes that encode inhibitors of apoptosis (Mcl-1, Bcl-xl), cell-cycle regulators (cyclins D1/D2, c-Myc), and inducers of angiogenesis (VEGF) [7–9]. Moreover, STAT3 has also been shown to play a role in the pathogenesis of breast cancer through its positive effects on invasion, stem cell expansion, and modulation of the environment [10].

Activated or tyrosine phosphorylated STAT3 (pSTAT3) is found in approximately 50 % of breast cancers, with the highest expression levels identified in the leading edge of tumors, in areas of lymphovascular invasion and in axillary lymph nodes [10–13]. Additionally, STAT3 has been shown to mediate immune suppression by inhibiting expression of pro-inflammatory cytokines and chemokines necessary for immune cell activation [14]. The role of pSTAT3 as a negative regulator of immune cell-mediated antitumor responses and as an oncogenic driver led us to hypothesize that pSTAT3 expression might be a predictor of resistance to trastuzumab therapy in patients with HER2-positive breast cancer.

## Methods

### Patients and samples

For our study we used samples and data from three main sources, Responsify, FinHer, and the TCGA repository. The Responsify dataset (as part of the FP7 EU consortium; grant number 278659; [15]) is composed of 108 HER2-positive early stage breast cancer samples treated with adjuvant trastuzumab provided by the Institut Jules Bordet and the Katholieke Universiteit Leuven. HER2 was defined by an immunohistochemistry (IHC) of 3+ or a positive result on fluorescence in situ hybridization (FISH) for HER2 amplification (HER2: Chr17 ratio ≥2). Gene expression data were available for 94 patients, reverse-phase protein lysate microarrays (RPPA) for 51 patients, and clinical outcome data for all patients. The clinical pathological characteristics of patients in the Responsify dataset are provided in Additional file 1: Table S1A. Study participants provided written informed consent to allow further research analyses to be carried out on their tumor tissue. Samples were accessed under Leuven Hospital ethics approval No. S52903. Samples were processed at the Institut Jules Bordet, Brussels, Belgium, under ethics approval no. EC1750.

The FinHer dataset was derived from the trial of the same name, a multicenter, phase 3, randomized breast cancer trial in the adjuvant setting that enrolled 1010 patients (CONSORT diagram Additional file 2: Figure S1) [1]. The women were randomly assigned to receive three cycles of docetaxel or vinorelbine, followed by three cycles of fluorouracil, epirubicin, and cyclophosphamide. Out of the 1,010 patients, 232 patients with HER2-positive breast cancers were further randomized to nine weeks of trastuzumab or to no trastuzumab. Overall, 202 of 232 HER2-positive tumors had sufficient good quality RNA for gene expression analysis.

The clinical pathological characteristics of the patients with HER2-positive tumors who also had available gene expression data (n = 202) were compared with the general series (n = 231) (Additional file 3: Table S1B). The patients with gene expression data were representative of the entire population, with no substantial differences in patient and tumor characteristics identified between the two groups (Additional file 3: Table S1B). Study participants provided written informed consent to allow further research analyses to be carried out on their tumor tissue. Gene expression profiling from the breast tumor samples was approved by the Helsinki University institutional review board (permission HUS 177/13/03/02/2011).

The primary end point of the FinHer study, distant disease-free survival (DDFS), has been previously reported to be superior for the trastuzumab-containing arms after follow-up of 62 months [1]. The determination of hormone receptor status and HER2 expression by IHC was required locally and was performed according to the guidelines of each institution during the time of the study. Samples were considered to be hormone receptor-positive if their level of estrogen receptor (ER) and/or progesterone receptor was ≥10 %. All patients with hormone receptor-positive tumors received 5 years of endocrine therapy. When HER2 expression was scored as 2+ or 3+ (on a scale of 0, 1+, 2+, or 3+), the number of copies of the HER2 gene was centrally determined by means of chromogenic in situ hybridization in reference laboratories.

### Gene expression arrays

The Responsify expression dataset was produced using the Affymetrix HG-U133Plus2 platform at the J-C Heuson Breast Cancer Translational Research Laboratory. Expression values were computed using the fRMA normalization method (fRMA Bioconductor package) [16]. From the Responsify dataset, a total of 97 samples corresponding to 95 unique patients were processed. Appropriate quality assessments were conducted on the resulting files, and 94 samples passed quality assurance for further analysis. The Responsify data is available at: [16].

As mentioned above, from the FinHer HER2 samples, gene mRNA expression data was produced from 202 samples. All samples were re-evaluated to ensure the tumor was present. RNA was extracted from formalin-fixed, paraffin-embedded primary breast tumor tissue. Gene expression was measured using Affymetrix U219 GeneChips™ as per Affymetrix protocols on 96 well plates at AROS applied biotechnology A/S, Denmark. Affymetrix expression data were normalized using the RMA approach, followed by a batch effect correction (Affy and SVA package of the Bioconductor suite) [17].

For both the Responsify and FinHer datasets, when multiple probe sets mapped to the same official gene symbol, we computed the average value.

In the TCGA repository, HER2-positive breast cancers were analyzed based on the RPPA phosphorylation levels [18]. Patients with a Z score greater than 0.2 (STAT3 high) or less than −0.2 (STAT3 low) in the overall distribution were selected for comparison using the cBioPortal web application [18] from Memorial Sloan Kettering Cancer Center [19]. mRNA expression data were also downloaded from the TCGA repository. In all cases, the downloaded data were TCGA level 3 [18]. The data (RPPA and gene expression) were uploaded to the GenePattern web software of the Broad Institute [20]. Patient data were analyzed for differential gene expression using the Comparative Marker Selection tool comparing STAT3 high and STAT3 low genes for each subtype (*P* = 0.001, fold ≥1). The TCGA data has open access through few portals and permission to access the TCGA data was not required.

The differences in platforms and methods across the different datasets, including the TCGA dataset that was used for external validation, are summarized in Additional file 4: Table S2 and Additional file 5: Figure S2D.

### Reverse phase protein array (RPPA)

The protein levels of the Responsify cohort were assessed in the laboratory of Gordon Mills at MD Anderson Cancer Center (Houston, TX) using RPPA, as previously described [18]. The following procedures were performed for the current RPPA core: tumor lysates were two-fold-serial diluted for five dilutions (from undiluted to 1:16 dilution) and arrayed on nitrocellulose-coated slide in 11 × 11 format. Samples were probed with antibodies by amplification approach and visualized by DAB colorimetric reaction. Slides were scanned on a flatbed scanner to produce 16-bit tiff image. Spots from tiff images were identified and the density was quantified by Array-Pro Analyzer. Relative protein levels for each sample were determined by interpolation of each dilution curves from the “standard curve” (supercurve) of the slide (antibody). All the data points were normalized for protein loading and transformed to linear value designated as “Linear after normalization”; 243 slides for 211 unique antibodies were stained and analyzed on Array-Pro then by supercurve R ×64 2.15.1. There were 14 sets of replicated antibodies and three negative controls for secondary antibodies among 243 slides. A quality control test was performed for each antibody staining (slide), in which a score above 0.8 indicates good antibody staining (all antibodies used in the present study).

### Computation of the pSTAT3 gene signature

To develop a predictive gene signature score, we computed the scalar product of the coefficient of the genes in the signature and the gene expression values (pSTAT3-GS). Fifty-one HER2-positive samples in the Responsify dataset with both available gene expression and RPPA data were evaluated. For the pSTAT3 RPPA assay, we considered two sample groups with clear “up” and “down” protein expression by splitting the samples to upper and lower quartile of the expression mean (Additional file 5: Figure S2A). To identify the genes that were differently expressed in the two groups, we performed gene expression analysis using a Student *t*-test (Welch adaptation with robust estimators of dispersion and central tendency) comparing high versus low tumors (false discovery rate (fdr) ≤0.05; Additional file 5: Figure S2B). To compute the expression of the pSTAT3-GS using available expression datasets, we defined a signature score: Let S be a gene signature composed of n genes (s1, …, sn) presenting a coefficient (–1 or 1, depending on the down-regulation or up-regulation, respectively) and let E be the set of expression values of the genes of S in one expression experiment.

The pSTAT3-GS score (Sigscore) is derived by computing the sum of the products of the gene coefficient in the module (S_i_) by the corresponding gene expression value (e_i_) according to the following formula:

$$ Sigscore={\displaystyle \sum_{i=1}^n{e}_i*{S}_i} $$

### Histological stromal reactivation evaluation

Evaluation of stromal reactivation was performed simultaneously by two pathologists (RS, GVdE) blinded to the molecular data. The methodology and cut-offs were pre-defined before the evaluation. Stromal reactivity was defined as scar-like, desmoplastic tissue containing a higher proportion of reactive myofibroblasts compared to the normal stroma of the breast, which does not contain reactive myofibroblasts. For the assessment of the stromal reactivity score, the proportion of the stromal area containing reactive myofibroblasts in proportion to the whole stromal area of both the invasive tumor area and the full slide (containing reactive stroma and normal non-reactive stroma) was evaluated. Tumors were divided into three groups on the basis of the amount of reactive stroma; Score 1: 0–30 % of myofibroblasts in 10 High Power-fields; Score 2: 31–60 % of myofibroblasts in 10 High Power fields, and score 3: 61–100 % of myofibroblasts in 10 High Power fields.

### Statistical analysis

For the outcome analysis, patients were scored according to the pSTAT3-GS. The primary outcome for the Responsify dataset was disease free survival (DFS) and for the FinHer, DDFS, which was defined by the time interval between the date of randomization and the date of first cancer recurrence outside of the ipsilateral/locoregional region or to death, whichever occurred first.

Patients alive at the last visit without documented evidence of distant metastases were censored. Associations between the pSTAT3-GS, pSTAT3, and clinical pathological parameters or other proteins were investigated with a *t*-test, and the correlation was estimated using a linear regression and a Pearson index. Survival curves were generated according to the Kaplan–Meier method, and survival distributions were compared using the log-rank test. Univariate and multivariate models were computed with Cox proportional hazards regression. The possible interaction with trastuzumab treatment was tested using a likelihood ratio test between Cox survival models adjusting or not for the trastuzumab treatment. Analyses were performed using the R statistical suite together with the Bioconductor ‘genefu’ package. The ability of the pSTAT3-GS to predict the pSTAT3 RPPA status of the patients was assessed with receiver operating characteristic (ROC) curves. RPPA pSTAT3-positive (mean +1SD) and pSTAT3-negative (mean +1SD) were used as cut-offs in HER2-positive breast cancers from the TCGA (N = 81). ROC curves were computed using the pROC library of the R statistical suite.

Significant associations between the signature scores and the stromal reactivation status were assessed using one-tailed Mann–Whitney tests comparing the signature scores in samples with low stromal reactivation (+) compared to samples with moderate or high (++/+++) stromal reactivation.

Our data were reported according to the essential elements of the “Reporting Recommendations for Tumor Marker Prognostic Studies (REMARK)” [21].

## Results

### pSTAT3 HER2-positive breast cancers are associated with a distinct gene expression profile

RPPA of pSTAT3 (tyrosine 705) was performed on 51 primary HER2-positive breast cancers using the Responsify dataset, which is composed of 108 HER2-positive early stage breast cancer patients treated with adjuvant trastuzumab (Additional file 1: Table S1A). No significant correlation was found between pSTAT3 status and any classic clinicopathological features (Additional file 1: Table S1A).

To determine whether pSTAT3 signaling pathway activation was associated with specific transcriptional changes, we compared gene expression profiles obtained from pSTAT3-positive (upper quartile) and pSTAT3-negative (lower quartile) samples using a two-sample *t*-test (Additional file 5: Figure S2A). Overall, 123 genes were significantly (positively or negatively) (fdr ≤0.05) and differentially expressed between pSTAT3-positive and pSTAT3-negative tumors, suggesting a distinct gene expression pattern associated with pSTAT3 signaling pathway activation (Dataset Additional file 5: Figure S2B, Additional file 6: Table S3).

To assess whether these genes could predict the phosphorylation status of STAT3, we computed a pSTAT3 gene expression signature score (pSTAT3-GS) according to their respective gene expression values. As expected, we found a positive significant correlation between the pSTAT3 RPPA and the pSTAT3-GS expression levels in the whole Responsify dataset, from which the signature was developed (r = 0.62, *P* = 0.19e^-6^).

To independently validate the ability of the pSTAT3-GS to determine pSTAT3 proteomic status in HER2-positive breast cancer, we used the TCGA cohort of patients with HER2-positive breast cancer in which gene expression and RPPA data are available [19]. As shown in Additional file 5: Figure S2C, the ability of the pSTAT3-GS to classify tumors based on their pSTAT3 proteomic status was significant, validating its predictive performance (area under the curve [AUC] of the receiver operating curve, 0.77; *P* = 0.02).

A gene set enrichment analysis [20] showed enrichment with Jak-STAT-regulated genes (*P* = 0.006; fdr = 0.14), as well as with genes associated with cell surface signal transduction and protein kinase activity. This suggests that the pSTAT3-GS captures pSTAT3 signaling pathway activation (Additional file 7: Table S4).

### Association of the pSTAT3-GS with clinical outcome in patients with HER2-positive breast cancer treated with trastuzumab

**Fig. 1:**
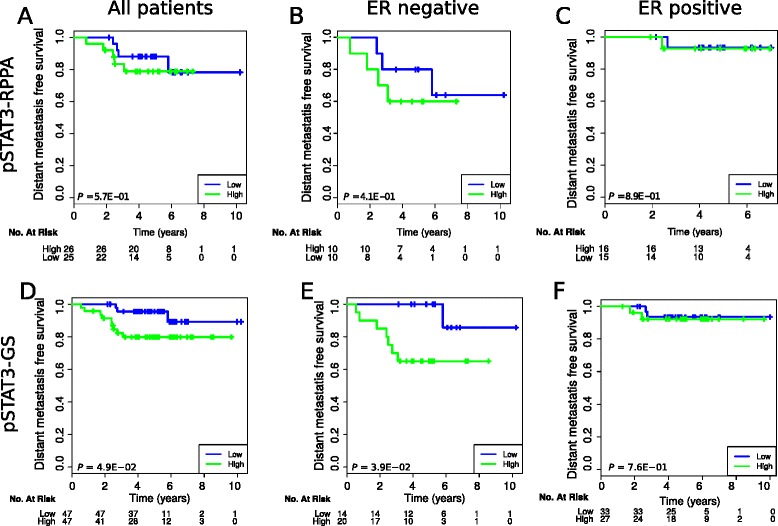
pSTAT3 RPPA and P-STAT3-GS survival analysis. **a**–**c** Kaplan–Meier curves according to pSTAT3 RPPA status in all patients (**a**), ER-negative (**b**), or ER-positive patients (**c**) for whom RPPA data was available in the Responsify dataset. **d**–**f** Kaplan–Meier curves according to pSTAT3-GS status in all patients (**d**), ER-negative (**e**), or ER-positive patients (**f**) for whom the gene expression data was available in the Responsify dataset

Since pSTAT3 has been reported to play a role in the pathogenesis of breast cancer through its positive effects on invasion, its modulation of the microenvironment, and its role as a negative regulator of immune cell–mediated antitumor responses, we hypothesized that pSTAT3 expression might influence response to trastuzumab therapy. To address this, we first correlated the phosphorylation status of pSTAT3 with clinical outcome using the Responsify dataset, in which all patients received trastuzumab in the adjuvant setting and for which we had RPPA available data (N = 51). No association was found between pSTAT3 protein levels and clinical outcome (Fig. 1a–c).

Since the numbers were small, we sought to interrogate whether the pSTAT3-GS, which mirrors STAT3 pathway activation, could predict clinical outcome on the same dataset involving a larger number of patients with available gene expression data (N = 94). Of note, the clinical outcome was not used to develop the pSTAT3-GS, and hence all the survival analyses were unbiased in their estimate of its performance. Interestingly, high pSTAT3-GS (dichotomized at the median) was significantly associated with poor outcome. This observation was mainly driven by the ER-negative subgroup (DFS, log rank *P* = 0.039 for ER-negative; Fig. 1d–f).

**Fig. 2:**
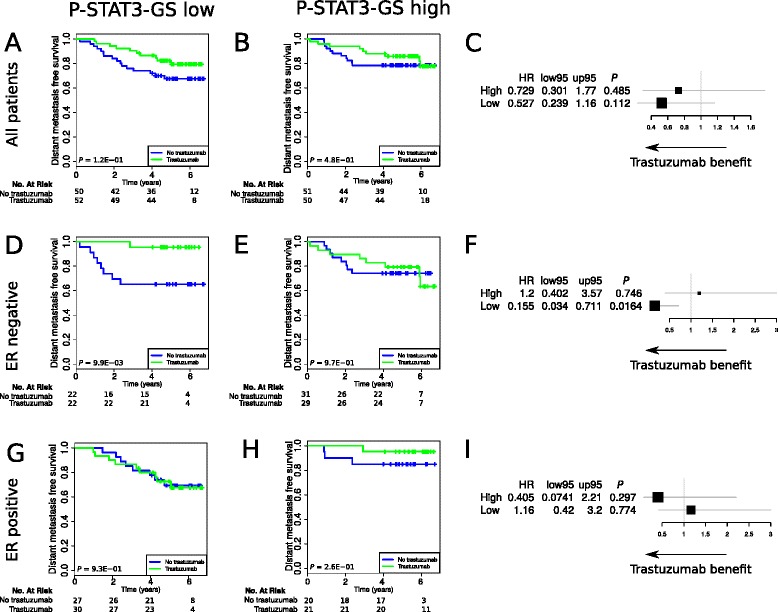
The pSTAT3-GS has predictive significance in the independent randomized FinHer dataset. **a**–**i** Kaplan–Meier curves and forest plots of signature status assessed in the FinHer dataset. Patients with pSTAT3-GS low (**a**, **d**, **g**) and pSTAT3-GS high (**b**, **e**, **h**) status according to trastuzumab treatment in all patients (up), ER-negative only (middle), or ER-positive only (down). Forest plots according to pSTAT3-GS status in all patients (**c**), ER- negative only (**f**), or ER-positive only (**i**). The plots indicate Cox regression hazard ratios and 95 % confidence intervals for trastuzumab benefit for DDFS

These observations were further validated in an independent dataset of patients treated in the prospective FinHer trial, in which patients were randomized to trastuzumab in the adjuvant setting (Additional file 3: Table S1B, CONSORT diagram S1). In this validation series, high pSTAT3-GS was associated with a lack of benefit from trastuzumab in the ER-negative subgroup when compared to low pSTAT3-GS (DDFS, *P* = 0.01; Fig. 2). Cox univariate and multivariable analysis of the pSTAT3-GS in the FinHer study confirmed – with a significant interaction test of *P* = 0.02 (Table 1) – that the pSTAT3-GS could provide independent predictive information for patients with ER-negative breast cancer who had been treated with trastuzumab. Overall, our data suggest that pSTAT3 pathway activation is predictive for trastuzumab resistance in HER2-positive/ER-negative breast cancer.

**Table 1:** Cox univariate and multivariable analysis of pSTAT3-GS treated as a continuous variable, in the FinHer study

	DDFS prognostic value of pSTAT3-GS (No trastuzumab)						DDFS prognostic value of pSTAT3-GS (trastuzumab)						
	Univariate			Multivariate			Univariate			Multivariate			*P* interaction
	HR	95 % CI	*P*	HR	95 % CI	*P*	HR	95 % CI	*P*	HR	95 % CI	*P*	
All	0.77	0.53–1.13	0.18	0.77	0.52–1.13	0.19	0.95	0.58–1.55	0.84	0.99	0.6–1.67	0.99	0.41
ER–	0.67	0.39–1.15	0.14	0.66	0.39–1.12	0.12	2.24	1.2–4.16	0.01	1.73	0.87–3.45	0.12	0.02
ER+	0.81	0.46–1.41	0.45	0.73	0.35–1.53	0.41	0.34	0.14–0.77	0.01	0.36	0.14–0.98	0.04	0.24

DDFS, Distant disease free survival; HR, Hazards ratio; CI, Confidence interval; ER, Estrogen receptor

For multivariate analysis, we considered the following variables: age, tumor size, grade, nodal status, and ER status (when ER was used to stratify the groups it was not used in the multivariate model). Interaction test for the multivariate analysis

### pSTAT3 is associated with PTEN loss and stromal reactivation

**Fig. 3:**
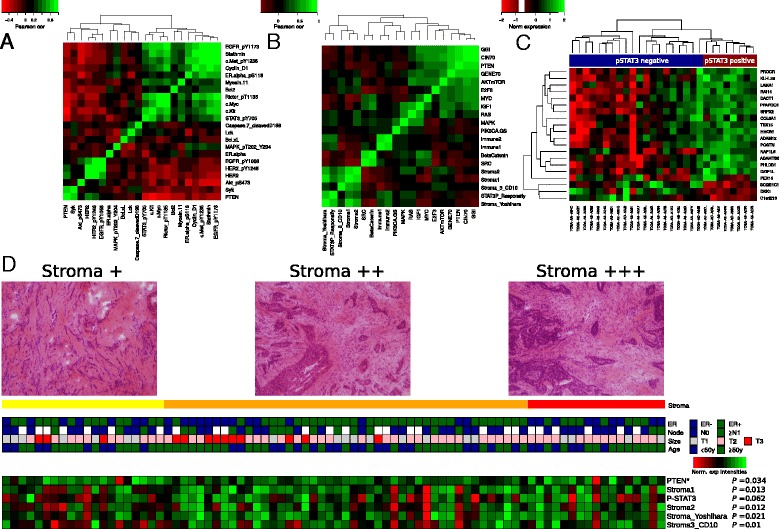
pSTAT3 and pSTAT3-GS are associated with PTEN loss and stromal reactivation. **a** pSTAT3 is associated with PTEN loss. Heatmap representation of the correlations between the RPPA values in the Responsify dataset. Cells are colored according to Pearson correlation coefficient values, with green indicating positive correlation and red negative correlations. **b** pSTAT3-GS is associated with stromal reactivation. The heatmap reflects the hierarchic clustering of pairwise correlations between different gene signatures in the Responsify dataset. Cells are colored according to Pearson correlation coefficient values, with green indicating positive correlation and red negative correlations. **c** The heatmap of the top significantly enriched genes in PAM50-identified patients with HER2-positive breast cancer annotated in the TCGA (*P* <0.001, fold >1), selected according to the high or low RPPA expression level of pSTAT3. Cells are colored according to the gene expression values, with green indicating positive correlation and red negative correlation. **d** pSTAT3-GS correlates with histological stromal reactivation. Histological sections showing breast tumors containing low (+), intermediate (++), and high (+++) reactive stroma. Heatmap shows correlation of reactive stromal content with clinical pathological parameters (not significant) and correlation with different gene signatures including stromal signature and pSTAT3-GS. * Negative correlation, *P* values were assessed using Mann–Whitney test

Considering studies that have suggested that STAT3 could participate in oncogenesis through the up-regulation of genes encoding cell-cycle regulators (cyclins D1, c-Myc), and a recent report of *in vitro* data suggesting that PTEN signaling may be associated with trastuzumab resistance [21], we sought to investigate whether there was any relationship at the protein level between pSTAT3, PTEN, and other proteins regulated by STAT3 in our clinical samples. Using RPPA (211 proteins) in the Responsify dataset, we found that pSTAT3 was negatively correlated with PTEN (r = –0.4, fdr = 0.025) and positively correlated with stathmin (r = 0.66, fdr = 0.03), a known marker of PTEN loss [22] (Fig. 3a). Other significant positive correlations with STAT3 included c-Myc (r = 0.39, fdr = 0.04), c-Kit (r = 0.66, fdr = 1.1e^-5^), and pEGFR (r = 0.52, fdr = 0.001). pcMET and cyclin D1 were also positively correlated, but did not pass the fdr ≤0.05 threshold. These data confirm that in primary HER2-positive breast cancer, STAT3 participates in oncogenesis through the up-regulation of genes encoding cell-cycle regulators and that PTEN loss may be associated with STAT3 activation.

Similar *in silico* analysis was performed at the gene expression level with the pSTAT3-GS and signatures involving different signaling pathways and biological processes [23]. As seen in Fig. 3b, the pSTAT3-GS was mainly correlated with several stromal signatures, suggesting a potential link between STAT3 expression and stromal activation [24–27]. Of note, the pSTAT3-GS was positively correlated with IL6 (r = 0.4, *P* = 4.72e^−05^), which is the principal cytokine pathway through which STAT3 is activated in breast cancer and a surrogate of stromal reactivation (Additional file 8: Figure S3).

Supportive findings have been reported for the TCGA dataset, in which HER2-positive breast cancers (based on the PAM50 classification) were analyzed according to their proteomic pSTAT3 status using the C-bio portal web server [23]. pSTAT3-positive tumors were associated with stromal reactivation genes, including high expression of POSTN, SRPX2, ADAM12, DACT1, and ADAMT6S (Fold >1, *P* <0.001; Fig. 3c).

Finally, to determine whether the pSTAT3-GS and stromal signatures are associated with histological-pathological stromal reactivation in HER2-positive patients, we performed a blinded examination of the Responsify frozen sections. For each sample, the proportions of reactive stroma were evaluated by two pathologists. Tumors were divided into three groups on the basis of the amount of reactive stroma. Comparison of the histological data showed a positive association of pSTAT3-GS and the different stromal gene signatures with the amount of reactive stroma, while the PTEN gene signature was negatively associated with reactive stroma (Fig. 3d).

Overall, these data suggest that there is a potential link between IL6-pSTAT3-PTEN loss, stroma reactivation, and primary trastuzumab resistance in HER2-positive primary breast cancers.

## Discussion

In this study, we integrated RPPA and gene expression data in order to interrogate the pSTAT3 signal transduction pathway in HER2-positive breast cancer. Specifically, we sought to determine whether constitutive STAT3 pathway activation could be responsible for primary resistance to trastuzumab in this breast cancer type. We observed that STAT3 phosphorylation was associated with a distinct gene expression signature for STAT3 pathway activation, and that this pSTAT3-GS was associated with trastuzumab resistance in two independent datasets. To our knowledge, ours is one of the first studies to use such a methodology and to demonstrate that an RPPA-based gene expression signature may reflect the proteomic activation status of samples. One of the strengths of our study is that it used gene expression data from a prospective clinical trial that randomized patients with HER2-positive breast cancer to receive treatment with or without trastuzumab.

Two recently published papers have used the general same approach to link upstream signaling pathways to downstream transcriptional response by exploiting RPPA and mRNA expression in breast cancer, demonstrating the robustness of this approach [28, 29].

A few studies have suggested that the activation of an IL6 inflammatory loop through STAT3 mediates trastuzumab resistance in HER2-positive breast cancer by expanding the cancer stem cell population and by promoting epithelial-mesenchymal transition [21, 30, 31]. While these studies focused on pre-clinical acquired resistance, in our study we provide evidence for primary resistance in the clinical adjuvant setting. Moreover, we identified STAT3 activation in a subset of patients with PTEN loss, suggesting that novel strategies to block the STAT3 pathway in combination with trastuzumab treatment may be especially relevant in PTEN- deleted breast cancer. The functional importance of IL6-STAT3 activation in PTEN-deleted HER2-positive breast cancer was demonstrated in a study showing that the IL6R antibody, alone or in combination with trastuzumab, decreased the cancer stem cell population and inhibited development of distant metastasis [21].

Recent studies have also highlighted the importance of the tumor micro-environment, such as stromal cells, in breast cancer prognosis and chemotherapy efficacy, particularly within the HER2-positive subgroup [24, 25, 27]. A mechanistic explanation for this phenomenon was demonstrated in an article showing that the IL6-STAT3 pathway drives tumor progression through the stroma and metastatic niche [31]. The positive correlation of IL6 and pSTAT3 with stromal reactivation signatures in our study supports the notion that this phenomenon occurs in HER2-positive human specimens. Given that levels of pSTAT3 are highest on the leading edge of tumors in association with stromal and endothelial cells, it is possible that samples that are pSTAT3-GS and stromal signature-positive in our study contain more material from the tumor edge or stroma. Indeed, the Yoshiara stromal signature [26], which represents the fraction of “normal” stromal cells in tumor tissue, also correlated positively with the pSTAT3-GS. Nevertheless, pSTAT3-GS still has the independent ability to predict resistance to trastuzumab.

In our study, the predictive role of pSTAT3 was confined to the ER-negative group while the ER-positive group showed an opposite pattern, although not significant. A possible explanation is that in the ER-positive group, different signal transduction pathways are involved in the activation of STAT3. Indeed, in a recent bioinformatics analysis, it was shown that low proliferating luminal breast cancers were much more likely to possess a high pSTAT3 phenotype [29]. In addition, analyzing tissue microarrays from breast cancer patients showed that pSTAT3 was associated with better prognosis [12, 13, 32]. These observations suggest that pSTAT3 may have different activators and targets in the different breast cancer subtypes. Although we used prospective randomized data in our study, the cohort from the FinHer trial was relatively small. We therefore acknowledge the need for further validation using a larger number of patients.

## Conclusions

**Fig. 4:**
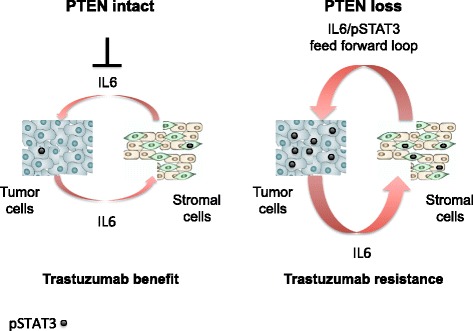
Model. There is a potential link between IL6-pSTAT3-PTEN loss, stromal reactivation, and primary trastuzumab resistance in HER2-positive primary breast cancers

Overall, we propose that the STAT3-stromal feed-forward loop, which can be enhanced by PTEN loss, is predictive of primary trastuzumab resistance (model; Fig. 4). If confirmed by future large prospective, randomized, controlled studies, inhibiting the IL6-STAT3 pathway [33] may be a valuable addition to trastuzumab treatment of primary HER2-positive breast cancer, especially those that are PTEN deficient.

## Additional files

Additional file 1: Table S1A. Responsify clinical and pathological data. Additional file 2: Figure S1. CONSORT diagram FinHer trial. Additional file 3: Table S1B. FinHer clinical and pathological data. Additional file 4: Table S2. Differences in platforms and methodologies between the different sets. Additional file 5: Figure S2. Schematic description of pSTAT3 gene signature building and assessment. (A) For 51 HER2-positive samples in the Responsify dataset we considered two sample groups with clear pSTAT3 “up” and “down” expression. (B) We compared gene expression profiles obtained from pSTAT3 positive (fourth quartile) and pSTAT3 negative (first quartile) samples using a two-sample *t*-test. Overall, 123 genes were significantly (fdr ≤0.05) and differentially expressed between pSTAT3-positive and pSTAT3-negative tumors. (C) ROC curve demonstrating predictive ability of the pSTAT3-GS as a continuous variable to predict pSTAT3 RPPA proteomic data in HER2-positive tumors in the TCGA repository. (D) Analysis of gene expression datasets comparing the genes (probes) of the STAT3 RPPA signatures from Responsify (blue) and genes whose expression were measured in the TCGA set (yellow) and the FinHer set (green). Additional file 6: Table S3. pSTAT3 gene signature. Additional file 7: Table S4. Enrichment analysis of the pSTAT3-GS genes when compared to Gene Ontology (GO) and oncogenic signatures available on the BROAD GSEA web server. Additional file 8: Figure S3. Scatter plot of the gene expression of IL6 as a function of the gene expression of the pSTAT3-GS signature in the Responsify dataset.

## Abbreviations

DDFS: Distant disease-free survival
DFS: Disease free survival
ER: Estrogen receptor
fdr: false discovery rate
pSTAT3: Phosphorylated STAT3
ROC: Receiver operating characteristic
RPPA: Reverse-phase protein lysate microarrays
STATs: Signal transducer and activator of transcription proteins
