# Supplementary material for: Constitutive phosphorylated STAT3-associated gene signature is predictive for trastuzumab resistance in primary HER2-positive breast cancer
Source: BMC Med. 2015 Aug 3;13:177. doi: 10.1186/s12916-015-0416-2 (PMC4522972; doi:10.1186/s12916-015-0416-2)
Supplement: Additional file 1: Table S1A. — Responsify clinical and pathological data. [file 12916_2015_416_MOESM1_ESM.docx]

**Table S1A Responsify clinical and pathological data**

|  |  | All eligible patients | RPPA analysed patients | pSTAT3-GS average score | pSTAT3 RPPA score (mean) | *P* |
| --- | --- | --- | --- | --- | --- | --- |
|  | No. of cases | 108 | 51 | 319.9 | 0.01 |  |
| Age | >=50 | 60 | 31 | 325.8 | -0.04 | 0.17 |
|  | <50 | 47 | 20 | 310 | 0.09 |  |
|  | mean | 52.37 | 53.57 |  |  |  |
| Tumor size | T0-2 | 25 | 9 | 312.9 | 0.05 |  |
|  | T2-5 | 63 | 32 | 319 | -0.02 |  |
|  | T>5 | 18 | 9 | 331.6 | 0.08 | 0.73 |
| Nodal status | N0 | 47 | 23 | 313.8 | -0.06 |  |
|  | N1-3 | 32 | 16 | 331.4 | 0.09 |  |
|  | N>4 | 23 | 9 | 310.9 | 0.039 | 0.39 |
| ER status | Positive | 67 | 31 | 315.8 | -0.002 |  |
|  | Negative | 40 | 20 | 326.2 | 0.02 | 0.75 |
