# Supplementary figures and images for: Constitutive phosphorylated STAT3-associated gene signature is predictive for trastuzumab resistance in primary HER2-positive breast cancer
Source: BMC Med. 2015 Aug 3;13:177. doi: 10.1186/s12916-015-0416-2 (PMC4522972; doi:10.1186/s12916-015-0416-2)

# CONSORT Diagram for the fin-her trial

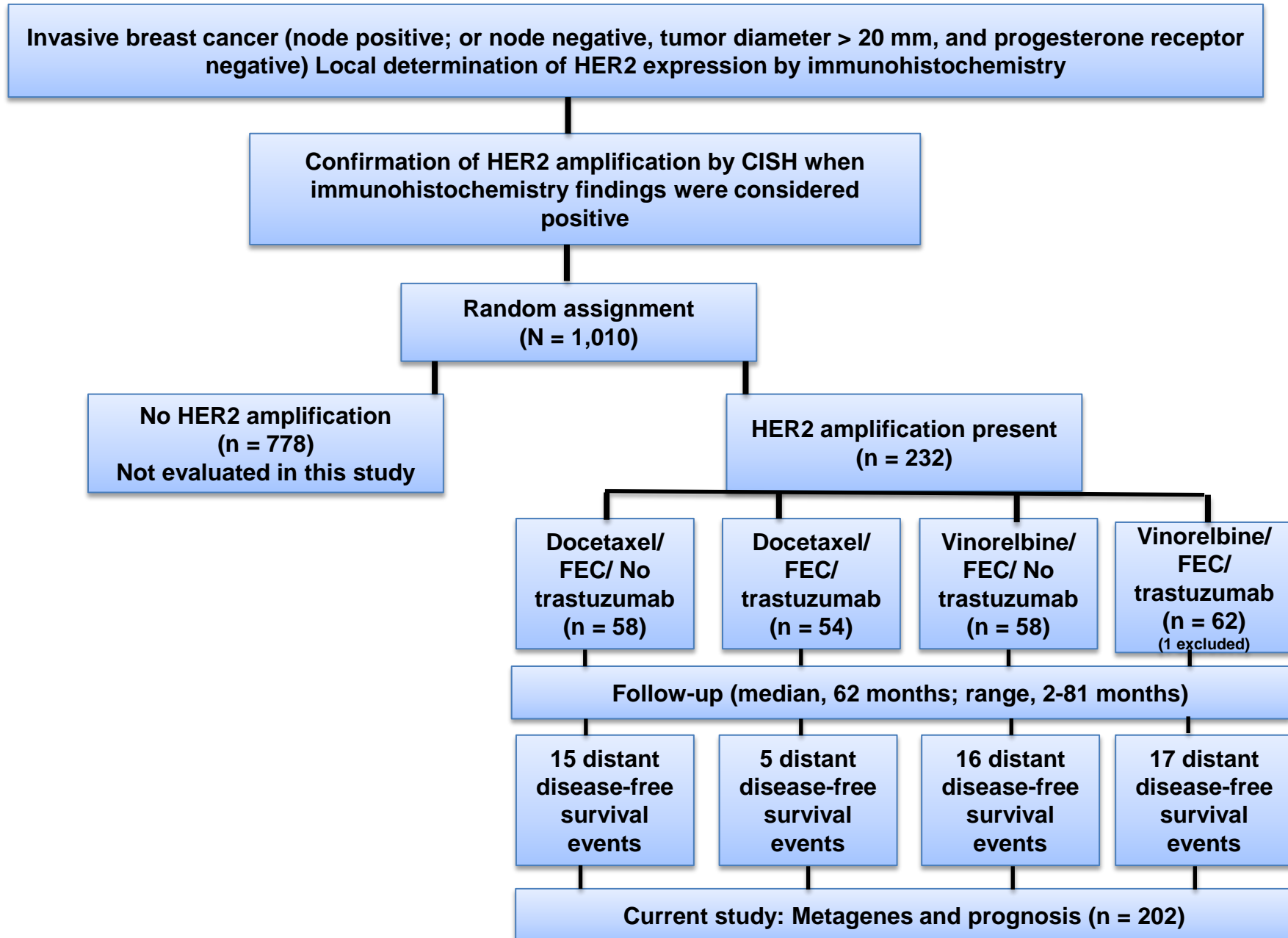

Supplement: Additional file 2: Figure S1. — CONSORT diagram FinHer trial. [file 12916_2015_416_MOESM2_ESM.pdf]

# Frequency

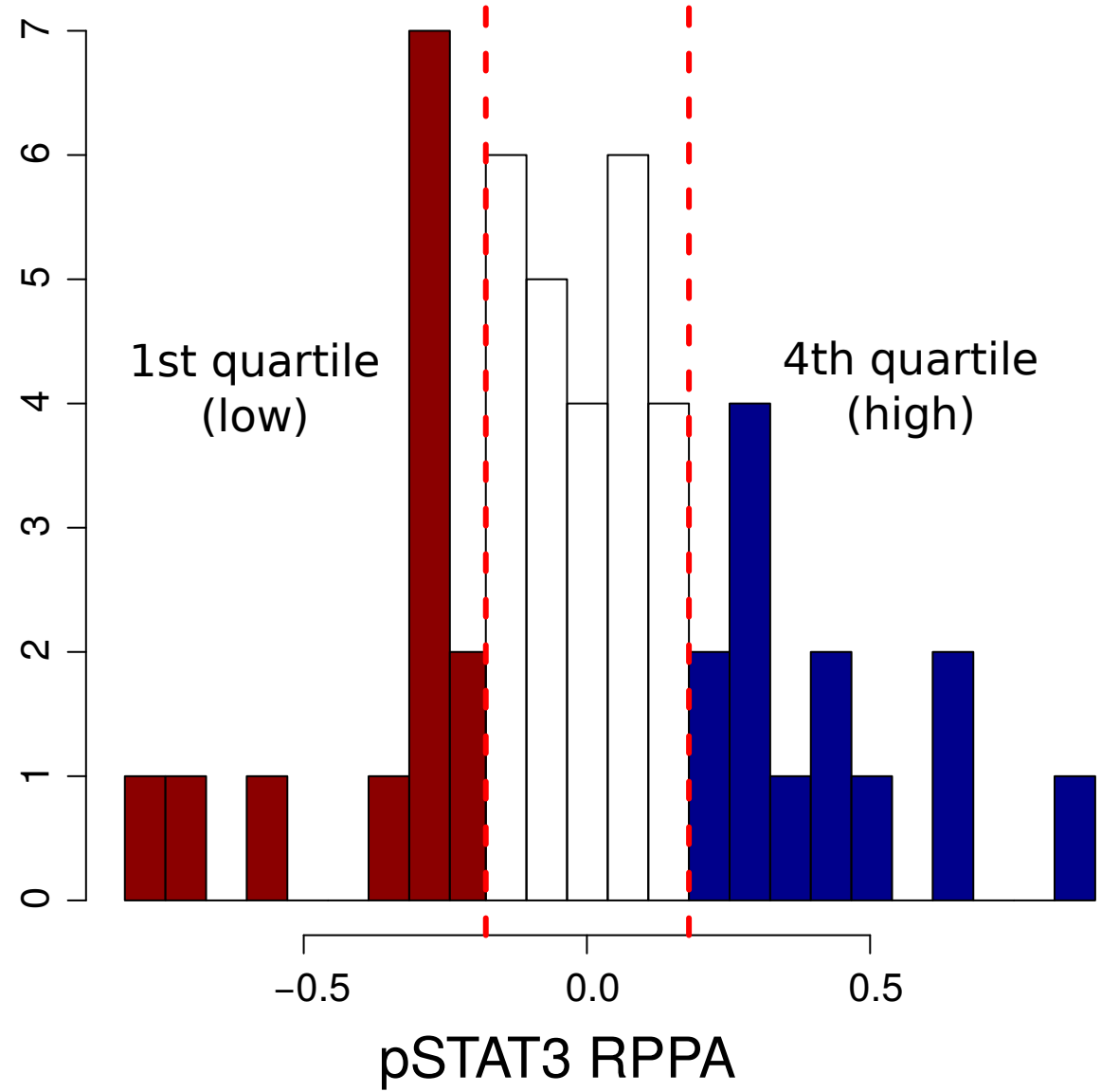

# B

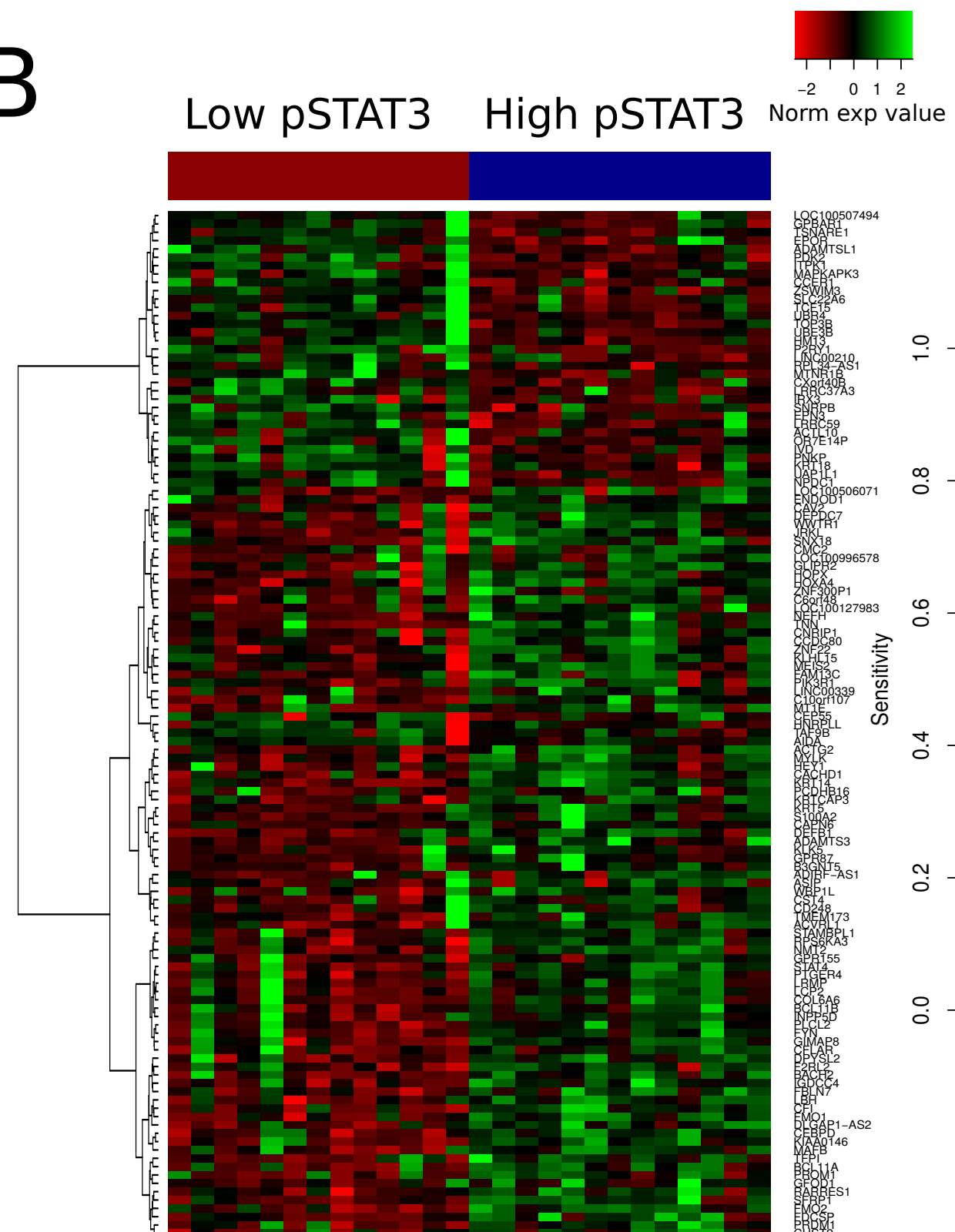

C

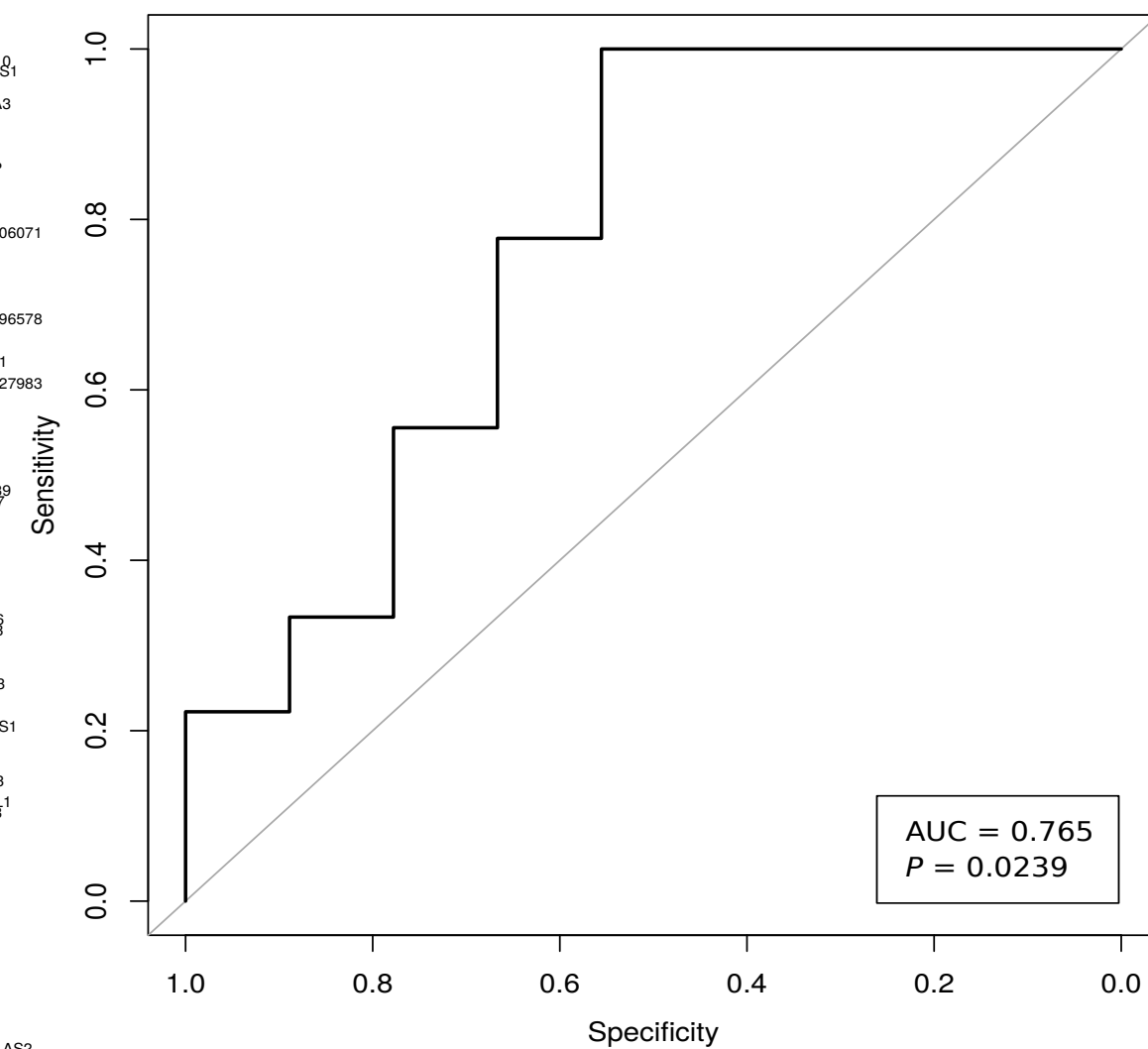

D

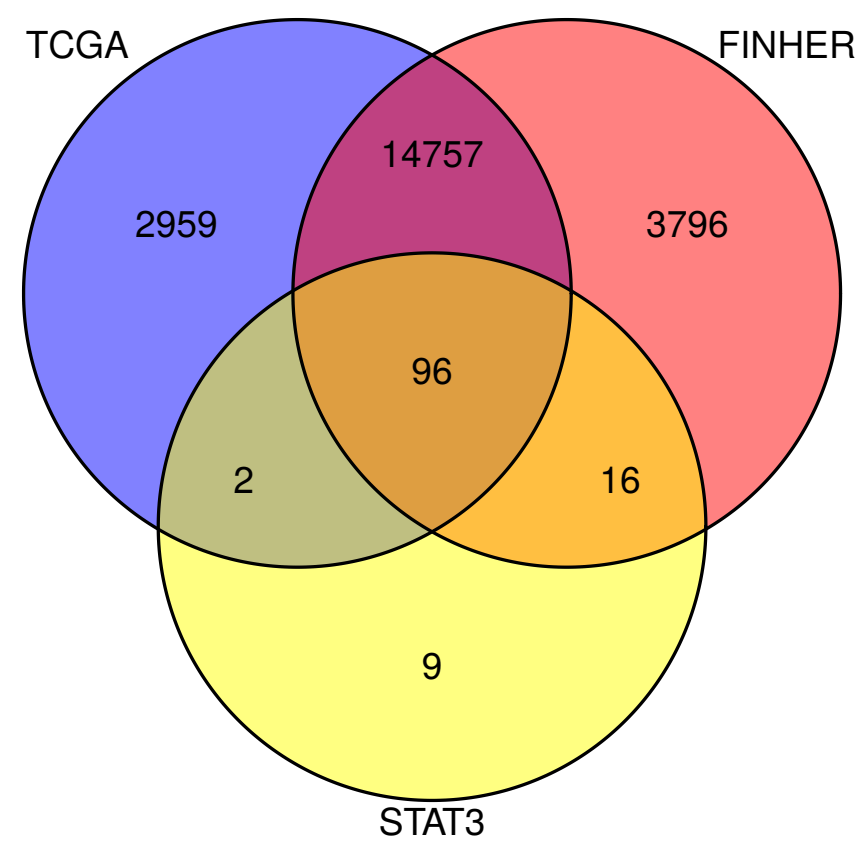

Supplement: Additional file 5: Figure S2. — Schematic description of pSTAT3 gene signature building and assessment. (A) For 51 HER2-positive samples in the Responsify dataset we considered two sample groups with clear pSTAT3 “up” and “down” expression. (B) We compared gene expression profiles obtained from pSTAT3 positive (fourth quartile) and pSTAT3 negative (first quartile) samples using a two-sample t-test. Overall, 123 genes were significantly (fdr ≤0.05) and differentially expressed between pSTAT3-positive and pSTAT3-negative tumors. (C) ROC curve demonstrating predictive ability of the pSTAT3-GS as a continuous variable to predict pSTAT3 RPPA proteomic data in HER2-positive tumors in the TCGA repository. (D) Analysis of gene expression datasets comparing the genes (probes) of the STAT3 RPPA signatures from Responsify (blue) and genes whose expression were measured in the TCGA set (yellow) and the FinHer set (green). [file 12916_2015_416_MOESM5_ESM.pdf]

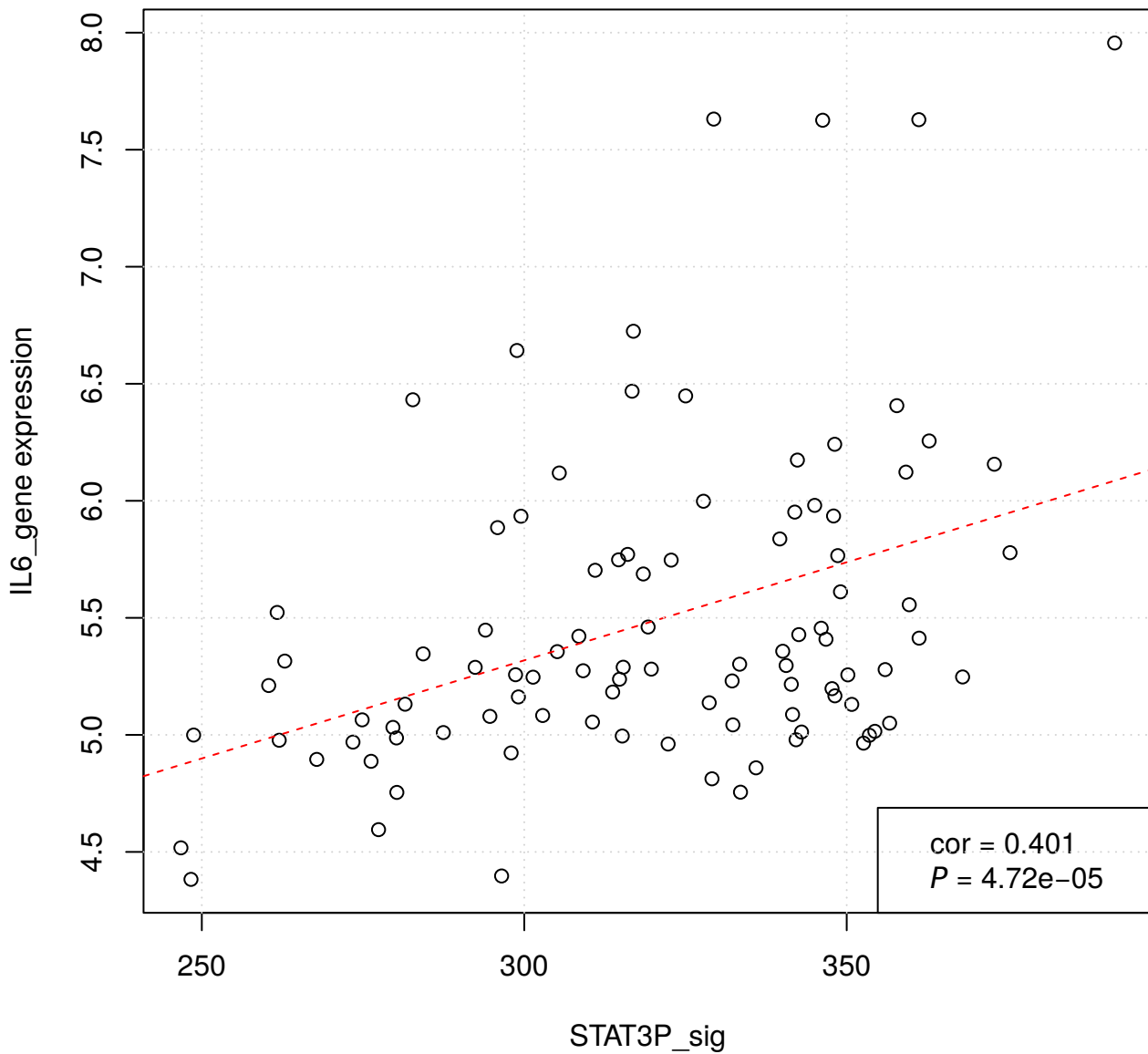

Supplement: Additional file 8: Figure S3. — Scatter plot of the gene expression of IL6 as a function of the gene expression of the pSTAT3-GS signature in the Responsify dataset. [file 12916_2015_416_MOESM8_ESM.pdf]
