# Supplementary material for: Constitutive phosphorylated STAT3-associated gene signature is predictive for trastuzumab resistance in primary HER2-positive breast cancer
Source: BMC Med. 2015 Aug 3;13:177. doi: 10.1186/s12916-015-0416-2 (PMC4522972; doi:10.1186/s12916-015-0416-2)
Supplement: Additional file 3: Table S1B. — FinHer clinical and pathological data. [file 12916_2015_416_MOESM3_ESM.docx]

| Characteristic | subclass | Whole HER2  positive cohort (N = 231) | Cohort with Gene expression (N=202) | P value | With trastuzumab (N=100) | No trastuzumab (N=102) | P value | P-STAT3-GS | P-value |
| --- | --- | --- | --- | --- | --- | --- | --- | --- | --- |
| Age | <50 | 108 | 94 | 1 | 51 | 43 | 0.26 | 166.5 |  |
|  | >=50 | 123 | 108 |  | 49 | 59 |  | 166.4 | 0.98 |
| Tumor stage | T1 | 81 | 74 | 0.94 | 33 | 41 | 0.49 | 166.8 |  |
|  | T2 | 135 | 115 |  | 60 | 55 |  | 166.2 |  |
|  | T3 | 14 | 12 |  | 7 | 5 |  | 165.7 |  |
|  | NA | 1 | 1 |  | 0 | 1 |  | 176.1 | 0.72 |
| Nodal status | Negative | 37 | 29 | 0.73 | 80 | 93 | 0.04 | 165.5 |  |
|  | 1-3 | 194 | 173 |  | 20 | 9 |  | 166.6 | 0.6 |
| Histological grade | 1 | 5 | 5 | 1 | 3 | 2 | 0.57 | 166.4 |  |
|  | 2-3 | 220 | 191 |  | 93 | 98 |  | 166.6 | 0.95 |
|  | NA | 6 | 6 |  | 4 | 2 |  | 163.0 |  |
| ER status | Positive | 109 | 97 | 0.93 | 46 | 51 | 0.66 | 164.6 |  |
|  | Negative | 122 | 105 |  | 54 | 51 |  | 168.2 | 0.041 |
| Histology | Ductal | 208 | 181 | 1 | 87 | 94 | 0.56 | 166.4 |  |
|  | Lobular | 21 | 19 |  | 11 | 8 |  | 168.9 | 0.39 |
|  | NA | 2 | 2 |  | 2 | 0 |  |  |  |

Table S1B. Fin-her clinical and pathological data
