# Supplementary material for: Constitutive phosphorylated STAT3-associated gene signature is predictive for trastuzumab resistance in primary HER2-positive breast cancer
Source: BMC Med. 2015 Aug 3;13:177. doi: 10.1186/s12916-015-0416-2 (PMC4522972; doi:10.1186/s12916-015-0416-2)
Supplement: Additional file 4: Table S2. — Differences in platforms and methodologies between the different sets. [file 12916_2015_416_MOESM4_ESM.docx]

| Data set | RNA expression dataset | Material | minimum Cellularity | Validation | RPPA | Antibody |
| --- | --- | --- | --- | --- | --- | --- |
| Responsify | Affymetrix platform HG-U133Plus2 | Frozen | 30% tumor cell nuclei | reviewed by a board-certified pathologist | MD Anderson | STAT3_pY705 |
|  |  |  |  |  |  | Cell signalling technology |
| Fin-her | Affymetrix U219 GeneChips™ | FFPE | 30% tumor cell nuclei | reviewed by a board-certified pathologist | N/A | N/A |
| TCGA | Agilent custom 244K whole genome microarrays | Frozen | 60% tumor cell nuclei | reviewed by a board-certified pathologist | MD Anderson | STAT3_pY705 |
|  |  |  |  |  |  | Cell signalling technology |

Table S2: Differences in platforms and methodologies between the different sets
