# Supplementary material for: Constitutive phosphorylated STAT3-associated gene signature is predictive for trastuzumab resistance in primary HER2-positive breast cancer
Source: BMC Med. 2015 Aug 3;13:177. doi: 10.1186/s12916-015-0416-2 (PMC4522972; doi:10.1186/s12916-015-0416-2)
Supplement: Additional file 6: Table S3. — pSTAT3 gene signature. [file 12916_2015_416_MOESM6_ESM.docx]

| name | coefficient |
| --- | --- |
| HM13 | -1 |
| ADAMTSL1 | -1 |
| EPOR | -1 |
| ITPK1 | -1 |
| CXorf40B | -1 |
| GPBAR1 | -1 |
| MAPKAPK3 | -1 |
| UAP1L1 | -1 |
| TSNARE1 | -1 |
| LRRC59 | -1 |
| IRX3 | -1 |
| EPN3 | -1 |
| NPDC1 | -1 |
| LINC00210 | -1 |
| ACTL10 | -1 |
| UBR4 | -1 |
| P2RY1 | -1 |
| TAF9B | -1 |
| PDK2 | -1 |
| SNRPB | -1 |
| TCF15 | -1 |
| CCER1 | -1 |
| AIDA | -1 |
| OR7E14P | -1 |
| CEP55 | -1 |
| HNRPLL | -1 |
| ZSWIM3 | -1 |
| TOP3B | -1 |
| UBE3B | -1 |
| LOC100507494 | -1 |
| LRRC37A3 | -1 |
| KRT18 | -1 |
| MTNR1B | -1 |
| PNKP | -1 |
| RPL34-AS1 | -1 |
| IVD | -1 |
| SLC22A6 | -1 |
| TNN | 1 |
| FMO2 | 1 |
| KIAA0146 | 1 |
| FYN | 1 |
| NEFH | 1 |
| SFRP1 | 1 |
| DEPDC7 | 1 |
| LOC100127983 | 1 |
| DEFB1 | 1 |
| FMO1 | 1 |
| KRT14 | 1 |
| LOC100506071 | 1 |
| PIK3R1 | 1 |
| BCL11A | 1 |
| SNX18 | 1 |
| FBLN7 | 1 |
| HEY1 | 1 |
| INPP5D | 1 |
| CACHD1 | 1 |
| HOPX | 1 |
| TMEM173 | 1 |
| DLGAP1-AS2 | 1 |
| CEBPD | 1 |
| GPR155 | 1 |
| CCDC80 | 1 |
| KRTCAP3 | 1 |
| STAT4 | 1 |
| B3GNT5 | 1 |
| KRT5 | 1 |
| RARRES1 | 1 |
| NMT2 | 1 |
| LCP2 | 1 |
| ASIP | 1 |
| MT1E | 1 |
| IGDCC4 | 1 |
| ADIRF-AS1 | 1 |
| ADAMTS3 | 1 |
| BACH2 | 1 |
| LRMP | 1 |
| LOC100996578 | 1 |
| COL6A6 | 1 |
| KLK5 | 1 |
| WWTR1 | 1 |
| LINC00339 | 1 |
| HOXA4 | 1 |
| C6orf48 | 1 |
| PTGER4 | 1 |
| STAMBPL1 | 1 |
| PCDHB16 | 1 |
| CAPN6 | 1 |
| ACVRL1 | 1 |
| FDCSP | 1 |
| ZNF300P1 | 1 |
| CAV2 | 1 |
| CD248 | 1 |
| CST4 | 1 |
| CHST2 | 1 |
| MAFB | 1 |
| CFI | 1 |
| MEIS2 | 1 |
| ACTG2 | 1 |
| CNRIP1 | 1 |
| BCL11B | 1 |
| MYLK | 1 |
| GFOD1 | 1 |
| DPYSL2 | 1 |
| F2RL2 | 1 |
| C10orf107 | 1 |
| FAM13C | 1 |
| GPR87 | 1 |
| WBP1L | 1 |
| RPS6KA3 | 1 |
| ZNF22 | 1 |
| GLIPR2 | 1 |
| ENDOD1 | 1 |
| PRDM1 | 1 |
| JRKL | 1 |
| GIMAP8 | 1 |
| S100A2 | 1 |
| CMC2 | 1 |
| CFLAR | 1 |
| KLHL15 | 1 |
| LBH | 1 |
| PROM1 | 1 |
| PLCL2 | 1 |
| TFPI | 1 |

Table S3. pSTAT3 gene signature
