# Supplementary material for: Constitutive phosphorylated STAT3-associated gene signature is predictive for trastuzumab resistance in primary HER2-positive breast cancer
Source: BMC Med. 2015 Aug 3;13:177. doi: 10.1186/s12916-015-0416-2 (PMC4522972; doi:10.1186/s12916-015-0416-2)
Supplement: Additional file 7: Table S4. — Enrichment analysis of the pSTAT3-GS genes when compared to Gene Ontology (GO) and oncogenic signatures available on the BROAD GSEA web server. [file 12916_2015_416_MOESM7_ESM.docx]

| Gene Set Name | # Genes in Gene Set (K) | # Genes in Overlap (k) | k/K | p-value | FDR q-value |
| --- | --- | --- | --- | --- | --- |
| ANATOMICAL_STRUCTURE_DEVELOPMENT | 1013 | 16 | 0.0158 | 2.79E-09 | 4.58E-06 |
| SYSTEM_DEVELOPMENT | 861 | 13 | 0.0151 | 1.60E-07 | 1.26E-04 |
| MULTICELLULAR_ORGANISMAL_DEVELOPMENT | 1049 | 14 | 0.0133 | 2.31E-07 | 1.26E-04 |
| SIGNAL_TRANSDUCTION | 1634 | 17 | 0.0104 | 3.61E-07 | 1.48E-04 |
| KRAS.LUNG_UP.V1_DN | 145 | 6 | 0.0414 | 1.45E-06 | 4.78E-04 |
| LTE2_UP.V1_UP | 190 | 6 | 0.0316 | 6.92E-06 | 1.89E-03 |
| ORGAN_DEVELOPMENT | 571 | 9 | 0.0158 | 1.01E-05 | 2.38E-03 |
| AKT_UP.V1_DN | 187 | 5 | 0.0267 | 9.16E-05 | 1.88E-02 |
| RAF_UP.V1_UP | 196 | 5 | 0.0255 | 1.14E-04 | 2.08E-02 |
| IMMUNE_SYSTEM_PROCESS | 332 | 6 | 0.0181 | 1.55E-04 | 2.15E-02 |
| PHOSPHOTRANSFERASE_ACTIVITY_ALCOHOL_GROUP_AS_ACCEPTOR | 334 | 6 | 0.018 | 1.60E-04 | 2.15E-02 |
| CELL_SURFACE_RECEPTOR_LINKED_SIGNAL_TRANSDUCTION_GO_0007166 | 641 | 8 | 0.0125 | 1.61E-04 | 2.15E-02 |
| NEGATIVE_REGULATION_OF_CELLULAR_PROCESS | 646 | 8 | 0.0124 | 1.70E-04 | 2.15E-02 |
| NEGATIVE_REGULATION_OF_BIOLOGICAL_PROCESS | 677 | 8 | 0.0118 | 2.33E-04 | 2.74E-02 |
| KINASE_ACTIVITY | 369 | 6 | 0.0163 | 2.73E-04 | 2.94E-02 |
| WOUND_HEALING | 54 | 3 | 0.0556 | 3.03E-04 | 2.94E-02 |
| CRX_DN.V1_DN | 134 | 4 | 0.0299 | 3.14E-04 | 2.94E-02 |
| NERVOUS_SYSTEM_DEVELOPMENT | 385 | 6 | 0.0156 | 3.42E-04 | 2.94E-02 |
| TISSUE_DEVELOPMENT | 138 | 4 | 0.029 | 3.51E-04 | 2.94E-02 |
| INTEGRAL_TO_MEMBRANE | 1330 | 11 | 0.0083 | 3.58E-04 | 2.94E-02 |
| INTRINSIC_TO_MEMBRANE | 1348 | 11 | 0.0082 | 4.00E-04 | 3.13E-02 |
| ATM_DN.V1_UP | 146 | 4 | 0.0274 | 4.35E-04 | 3.25E-02 |
| TRANSFERASE_ACTIVITY_TRANSFERRING_PHOSPHORUS_CONTAINING_GROUPS | 424 | 6 | 0.0142 | 5.69E-04 | 4.01E-02 |
| INTEGRAL_TO_PLASMA_MEMBRANE | 977 | 9 | 0.0092 | 5.86E-04 | 4.01E-02 |
| PROTEIN_KINASE_ACTIVITY | 285 | 5 | 0.0175 | 6.38E-04 | 4.09E-02 |
| INTRINSIC_TO_PLASMA_MEMBRANE | 991 | 9 | 0.0091 | 6.48E-04 | 4.09E-02 |
| EPIDERMIS_DEVELOPMENT | 71 | 3 | 0.0423 | 6.77E-04 | 4.12E-02 |
| CSR_LATE_UP.V1_DN | 170 | 4 | 0.0235 | 7.69E-04 | 4.51E-02 |

Table S4: Enrichment analysis of the pSTAT3-GS genes when compared to Gene Ontology (GO) and oncogenic signatures available on the BROAD GSEA web server. In red sets with hypothetical relevance to the signature
